# Thermo-hygrometric dynamics reveal divergent adaptive strategies in africanized and buckfast honey bees

**DOI:** 10.1007/s00484-026-03239-9

**Published:** 2026-05-22

**Authors:** Leandro Alves da Silva, Mateus Rodrigues Oliveira, Tasyely Daylhany Freire de Lima, Herica Girlane Tertulino Domingos, Genevile Carife Bergamo, Robson Mateus Freitas Silveira, Tamiles Araujo Lima, Débora Andrea Evangelista Façanha, Katia Peres Gramacho

**Affiliations:** 1 Postgraduate Program in Animal Science, Federal Rural University of the Semi-Arid, Mossoró, RN Brazil; 2 Department in Animal Science, Federal Rural University of the Semi-Arid, Mossoró, RN Brazil; 3 Animal Science Departament, State University of Vale do Acaraú, Sobral, Ceará Brazil; 4 Department of Natural Sciences, Mathematics and Statistics, Federal Rural University of the Semi-Arid, Mossoró, RN Brazil; 5 Department in Animal Science, “Luiz de Queiroz” Agriculture College (ESALQ), University of São Paulo, Piracicaba, São Paulo Brazil; 6 Institute of Culture and Art, Federal University of Ceará, Fortaleza, CE Brazil; 7 Department in Animal Science, Semi-Arid Rural Federal University, Mossoró, RN Brazil

**Keywords:** *Apis mellifera*, Heat stress, Thermoregulation, Semiarid

## Abstract

The present study investigated the adaptive strategies of Africanized and Buckfast honey bee colonies in a dry tropical environment, considering the thermohygrometric dynamics of the colonies. The internal temperature (IT) and internal relative humidity (IRH) were continuously recorded 24 h a day for seven months, yielding 36,360 observations in ten Africanized colonies and 22,680 observations in eight Buckfast colonies for each variable. Canonical discriminant analysis captured 100% of the data variance (*P* < 0.05) and revealed two significant functions (*p* < 0.001). Function 1 (81.8%) differentiated colonies along the IRH gradient, whereas Function 2 (18.2%) distinguished genotypes based on IT. For Africanized colonies, principal component analysis retained two components (75.37% of the variance, *P* < 0.05), with the first associated with the thermal–environmental axis and the second with IRH. Africanized bees exhibited greater thermo-hygrometric stability throughout the day, whereas Buckfast colonies showed more pronounced afternoon increases in IT and higher hygrometric fluctuations. This study compared the thermo-hygrometric dynamics of Africanized and Buckfast bees and revealed their distinct adaptive strategies. Buckfast bees relied more intensely on evaporative thermolysis, displaying lower thermal stability and higher humidity, whereas Africanized bees maintained thermal stability with reduced humidity variation, primarily using ventilation and secondarily, evaporative cooling associated with fanning. These findings demonstrate that physiological plasticity under continuous heat stress is critical for colony survival in dry tropical regions. Future studies should integrate these variables with productivity metrics and investigate the dynamics of colonies exposed to direct solar radiation.

## Introduction

Bees play a crucial role in maintaining biodiversity and food production worldwide; however, these pollinators face numerous biotic and abiotic stressors that compromise colony homeostasis, including the direct effects of climatic conditions (Potts et al. 2016; Gonzalez et al. 2024). For *Apis mellifera*, the challenge is even greater in hot and dry environments because they must dissipate heat while simultaneously preventing critical moisture loss within the colony. Thermoregulation through water evaporation becomes an energetically costly task in a context often marked by water scarcity, which tests the physiological and behavioral efficiency of colonies (Bonoan et al. 2014; Ma et al. 2021; Domingos et al. 2018, 2022).

Within the species *A. mellifera*, there is remarkable adaptive divergence between European honey bees and Africanized honey bees. European honey bees, evolutionarily adapted to temperate climates, developed strategies to conserve heat, such as forming clusters of workers combined with thoracic muscle shivering (Simpson 1991; Kutby et al. 2024), in addition to maintaining energy reserves that allow them to survive long periods of cold (Winston 1991; Abou-Shaara et al. 2017). In contrast, Africanized honey bees, with their strong ancestry from *A. m. scutellata* (Nelson et al. 2017), evolved in a tropical environment, which granted them high heat tolerance (Domingos et al. 2018; Poot-Báez et al. 2020) and reproductive strategies focused on rapid resource exploitation, even under geographically inhospitable conditions that pose significant survival challenges.

However, it is necessary to understand how European honey bees behave in dry tropical environments so that management strategies can be tailored to meet their specific needs, given that practices designed for Africanized honey bees may not be adequate for Buckfast bees. Thus, understanding thermoregulatory dynamics to develop individualized strategies that consider the evolutionary history of each group is a means of conserving these insects.

Although thermoregulation in European honey bees and Africanized honey bees is well documented, most comparative studies tend to focus solely on temperature (Toledo and Nogueira-Couto 1999; Poot-Báez et al. 2020), disregarding variations in colony relative humidity (and other environmental factors), which are essential for egg hatching and larval and pupal hydration (Kuhnholz and Seeley 1997; Human et al. 2006; Ostwald et al. 2016). These aspects highlight the need to consider thermo-hygrometric dynamics in an integrated manner throughout the colony’s daily and seasonal cycles to gain a deeper understanding of the adaptive strategies these bees may employ when managed under high temperatures and intense solar radiation.

## Materials and methods

The experiment was conducted over seven months (February to August 2024) in Mossoró, Rio Grande do Norte, Brazil (5° 4.116’ S; 37° 24.350’ W; 16 m above sea level). Ten Africanized honey bee colonies with queens naturally mated with Africanized drones and eight Buckfast colonies with queens mated in commercial apiaries in Argentina were used. All colonies were housed in Langstroth hives and maintained in an apiary with artificial shading (coconut leaf straw roofing). Sixty days before the experiment began, Buckfast queens were introduced into orphaned Africanized honey bees colonies. The colonies had similar conditions regarding population size, feeding, and health status as described above. A water reservoir located 50 m from the apiary provided a water source for foraging bees.

Inside the hives, internal temperature and internal relative humidity were recorded daily, 24 h per day, at sixty-minute intervals starting at 00:00, using RC-4HC dataloggers (Elitech-Brazil) placed in the central portion of each hive. The dataloggers were removed only on the data-reading days. Meteorological variables were obtained from an automatic weather station located 300 m from the apiary. The variables included air temperature (°C), relative humidity (%), rainfall (mm), and solar radiation (W/m²).

The relationship between the variables (Internal Temperature and Internal Relative Humidity) and groups (genotype × month) was evaluated using canonical discriminant analysis, applying the stepwise method to select variables with greater discriminant power. The significance of the functions was tested using Wilks’ lambda (λ) (*P* < 0.05). The proportion of variance explained was indicated by eigenvalues and canonical correlations, and the standardized coefficients revealed the contribution of each variable. Principal component analysis was conducted to explore the relationships among variables in a multivariate context. Data were analyzed using the Statistical Package for the Social Sciences (SPSS), version 20, 2010 (SPSS^®^ Inc., Chicago, IL, USA).

## Results

**Fig. 1:**
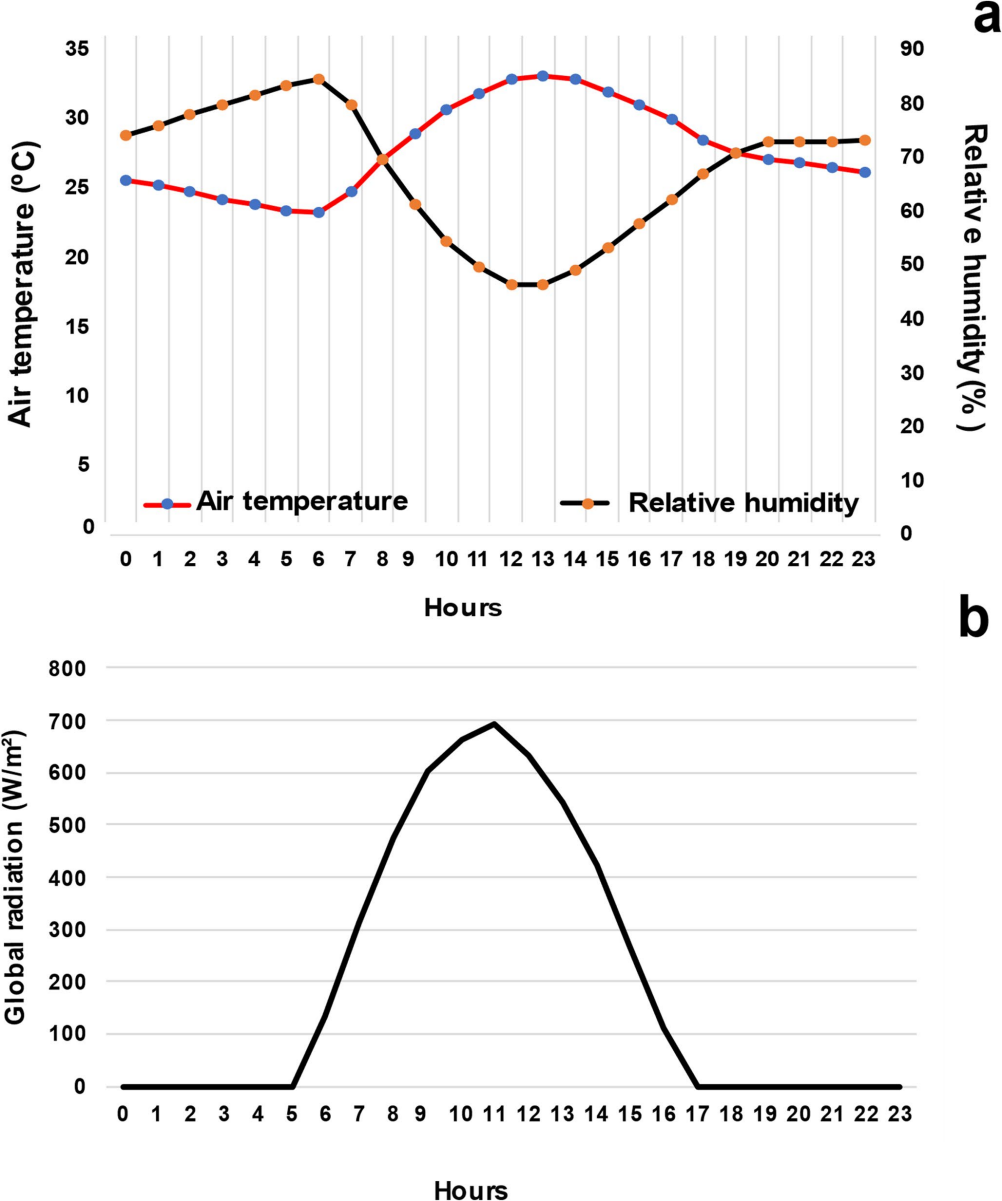
Mean values of air temperature (ºC), relative air humidity (%), and global solar radiation (W/m²) observed in Mossoró, RN, Brazil, from February to August 2024

Figure 1 presents the mean values of the environmental variables across 24 h during the seven months of collection. The mean air temperature, relative humidity (Fig. 1a), and solar radiation (Fig. 1b) were 27.8 °C, 67.3%, and 644 W/m², respectively; however, they reached maximum values of 38.9 °C at 14:00, 93.9% at 07:00, and approximately 1000 W/m² of solar radiation at 11:00.

According to the canonical discriminant analysis (Table 1), two canonical functions were significant in discriminating the groups (genotype and month) (Function 1: λ = 0.353, χ² = 61,383.5, df = 26, *p* < 0.001; Function 2: λ = 0.786, χ² = 14,215.5, df = 12, *p* < 0.001). Function 1 was primarily represented by internal relative humidity (coefficient = 1.066), with a secondary contribution from the internal temperature (coefficient = 0.375). Function 2 was explained mainly by internal temperature (coefficient = 0.998), with a minimal contribution from internal relative humidity (coefficient = − 0.007). The stepwise procedure indicated that both variables entered the final model, with internal relative humidity entering first, followed by internal temperature, which maintained a high F-value, confirming its complementary relevance to this model.

**Table 1:** Canonical discriminant analysis of thermo-hygrometric variables

Eigenvalues													
Function		Eigenvalue		Variance (%)		Cumulative %						Canonical correlation	
1		1.223ª		81.8		81.8						0.742	
2		0.272ª		18.2		100						0.463	
Wilks’ Lambda													
Test of Functions			**Wilks’ Lambda**				**Chi-square**		**df**			**Significance**	
1 to 2			0.353				61,383.524		26			< 0.001	
2			0.786				14,215.507		12			< 0.001	
Standardized canonical discriminant function coefficients													
						**Function 1**				**Function 2**			
Internal temperature (ºC)						0.375				0.998			
Internal humidity (%)						1.066				−0.007			
Variables not included in the analysis													
Step	**Variables**				**Tolerance**			**Minimum tolerance**			**F to enter**		**Wilks’ Lambda**
0	Internal temperature (ºC)				1.000			1.000			1,236.472		0.786
	Internal relative humidity (%)				1.000			1.000			5,019,303		0.475
1	Internal temperature (ºC)				0.880			0.880			1,559.918		0.353

**Fig. 2:**
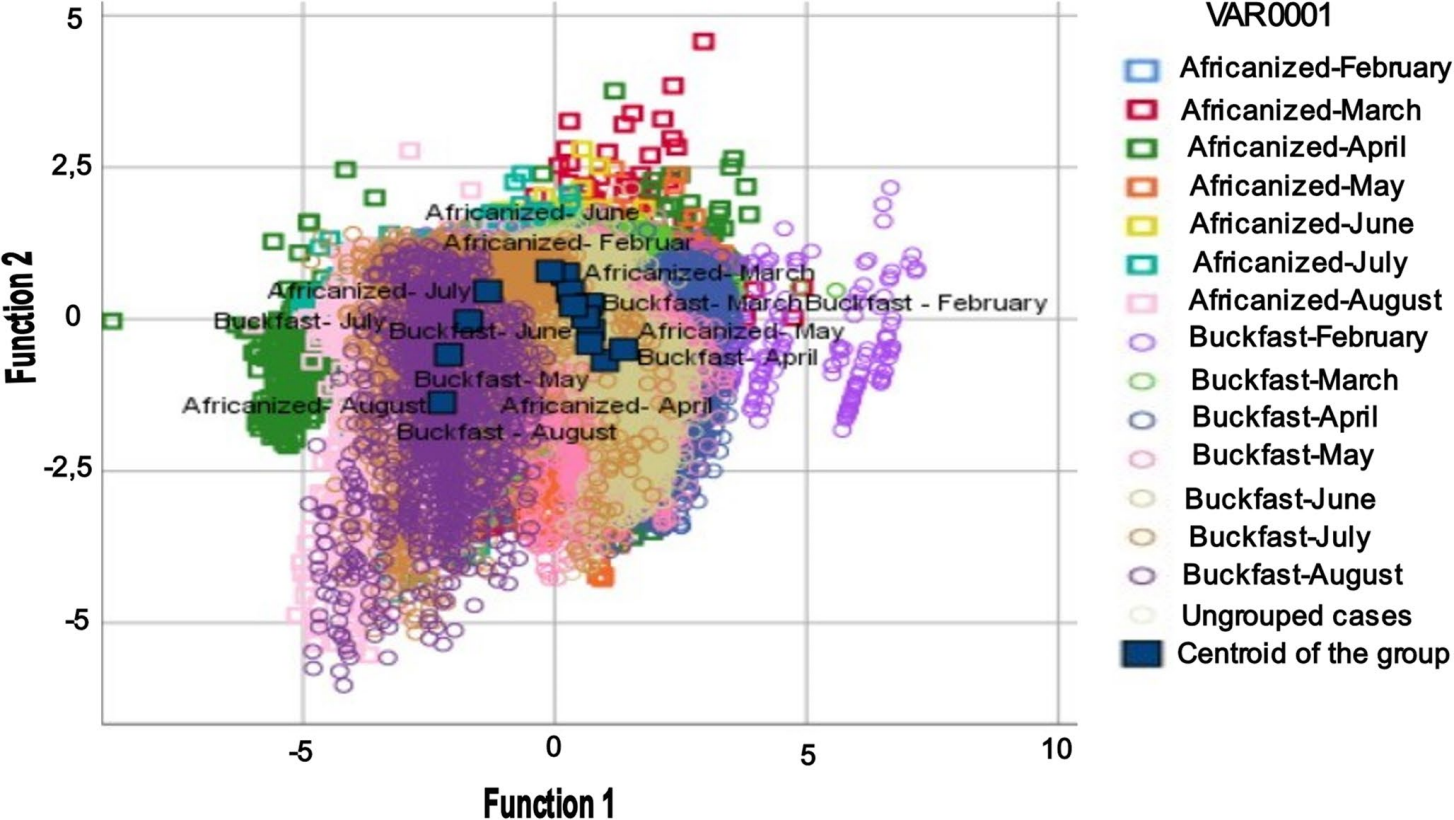
Canonical discriminant analysis biplot for the hive microclimate of Africanized and Buckfast honey bees in a semiarid environment

The classification dynamics of the colonies into their respective groups were represented by two canonical discriminant functions (Function 1: 81.8% of the variance, canonical correlation = 0.742; Function 2: 18.2% of the variance, canonical correlation = 0.463) (Fig. 2).

These functions showed partial separation among the groups, with Function 1 horizontally distributing the groups according to the internal relative humidity. Months in which colonies exhibited higher internal relative humidity (February to June for both genotypes) showed positive scores, whereas months with lower humidity (July and August for both genotypes) had negative scores. Function 2 differentiated the groups vertically based on internal humidity, with negative scores corresponding to below-average values (Buckfast: April, May, and August; Africanized: August).

**Fig. 3:**
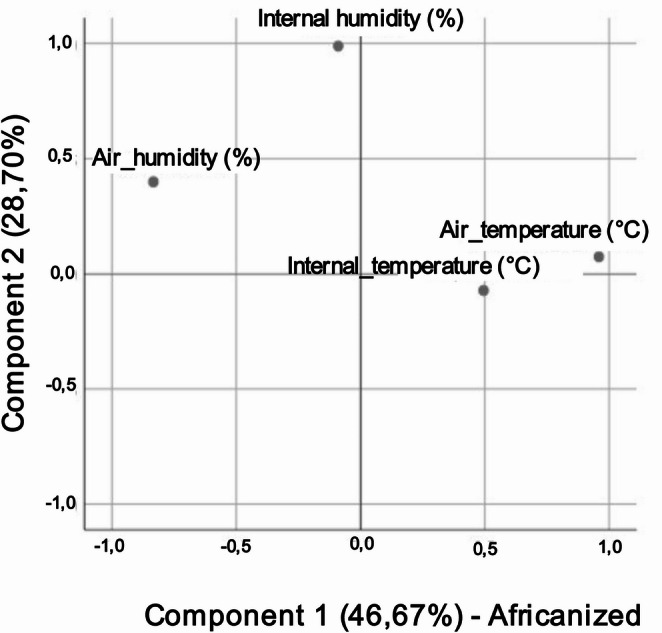
Exploratory factor analysis biplot of the seasonal adaptive dynamics of the microclimate in Africanized bee hives in a dry tropical environment

The principal component analysis for Africanized bees retained two components that explained 75.37% of the variance (Fig. 3). Component 1 (46.67%) was positively aligned with air temperature and internal temperature and negatively aligned with air humidity, characterizing a thermal–environmental axis. Component 2 (28.70%) aligned with the internal relative humidity, with minimal contribution from the thermal–environmental axis (Fig. 3).

**Fig. 4:**
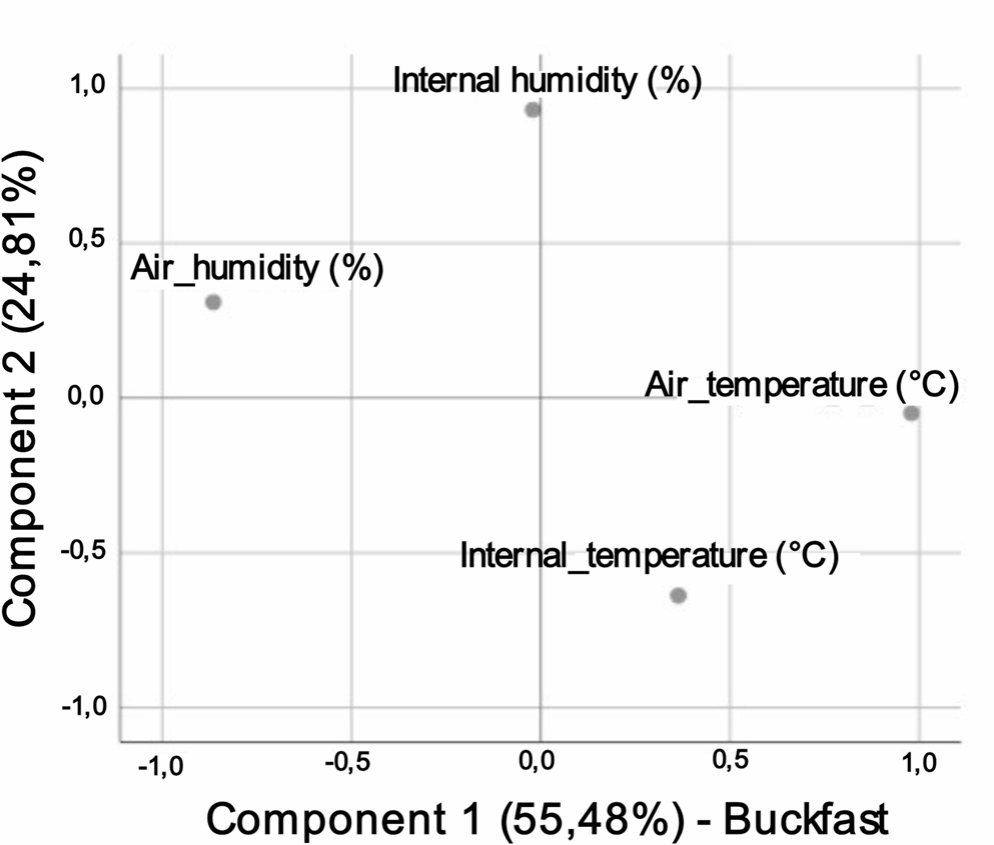
Exploratory factor analysis biplot of the seasonal adaptive dynamics of the microclimate in Buckfast bee hives in a dry tropical environment

For Buckfast bees, two components explained 80.29% of the variance (Fig. 4). Component 1 (55.48%) also represented a thermal gradient similar to that observed for Africanized honey bees, whereas Component 2 (24.81%) was primarily explained by internal relative humidity, with internal temperature showing a stronger negative loading, indicating a trade-off between the internal humidity and temperature.

**Fig. 5:**
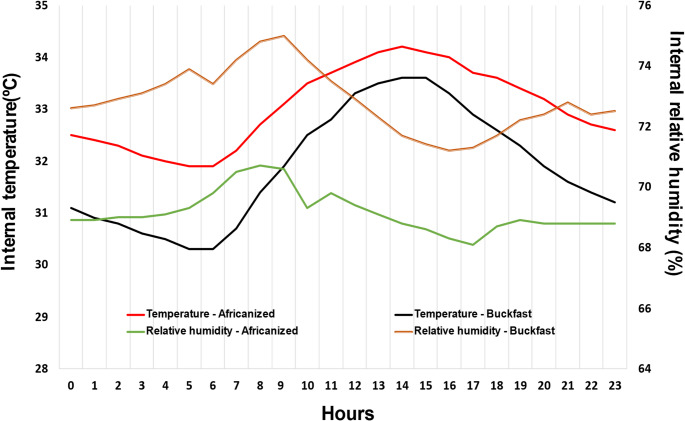
Mean internal temperature (ºC) and humidity (%) values of Africanized and Buckfast bee colonies evaluated 24 h per day between February and August 2024

The mean internal temperature and internal relative humidity values over the 24-hour cycle are shown in Fig. 5. Africanized colonies had mean internal temperature values of 33.0 °C, whereas Buckfast colonies averaged 32.0 °C. The mean internal relative humidity values were 69.0% for Africanized bees and 73.0% for Buckfast bees, with variations across hours.

**Fig. 6:**
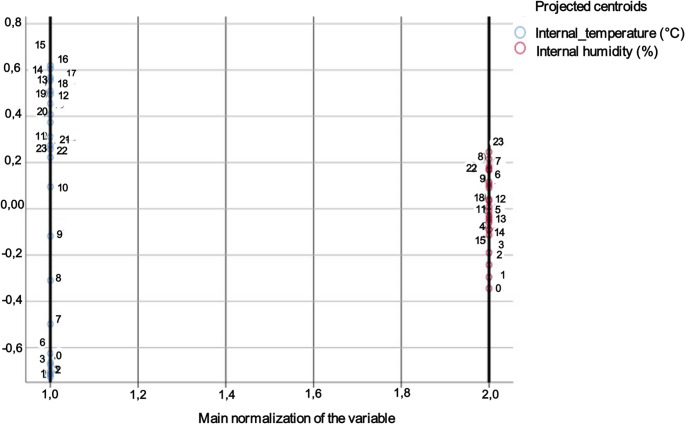
Daily seasonal adaptive dynamics of the hive microclimate in Africanized bee colonies in a dry tropical environment

Analysis of the thermo-hygrometric dynamics inside the hives over a 24-hour period revealed distinct, yet anticorrelated, daily patterns of internal temperature and internal relative humidity in both genotypes. In Africanized bees (Fig. 6), internal temperature remained above the daily mean from 10:00 to 20:00, peaking between 12:00 and 16:00. The lowest internal temperature values occurred between 00:00 and 06:00, remaining slightly negative until approximately 08:00 h. The internal relative humidity displayed the opposite pattern, with values above the mean concentrated between 22:00 and 09:00, peaking between 07:00 and 09:00. Lower humidity was observed between 14:00 and 17:00, increasing again after 18:00–19:00.

**Fig. 7:**
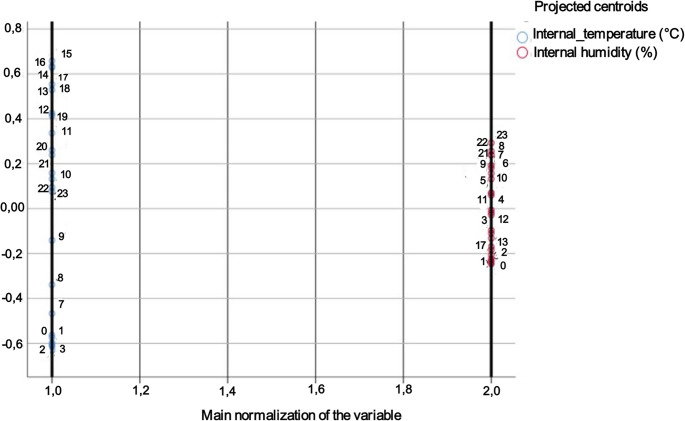
Daily seasonal adaptive dynamics of the hive microclimate in Buckfast bee colonies in a dry tropical environment

In Buckfast colonies (Fig. 7), internal temperature showed a more concentrated increase between 13:00 and 17:00, peaking between 14:00 and 16:00, followed by a faster return to the mean in the early evening (approximately 19:00–20:00). Minimum values occurred between 02:00 and 03:00, remaining below the mean until approximately 08:00–09:00. The internal relative humidity exhibited an inverse pattern relative to temperature, remaining above the mean from late night through the morning (approximately 20:00–09:00) and below the mean during the mid-afternoon period (approximately 12:00–17:00), with a rapid recovery around 19:00–20:00.

## Discussion

The present study investigated the adaptive differences between Africanized and Buckfast bees in a dry tropical environment, considering the thermo-hygrometric dynamics of the colonies. The external environment exerted strong control over the hive conditions, particularly through the thermal axis, which is characterized by high daytime temperatures and low relative humidity. This axis represents the set of external forces that act as the primary triggers for thermoregulation, determining when bees must activate the physiological and behavioral mechanisms of thermal control. Under such conditions, air temperature functions as the first warning signal, whereas relative humidity is a direct consequence of the adaptive strategies employed (Human et al. 2006) to mitigate thermal stress (Domingos et al. 2018, 2022; Santos et al. 2023; Silva et al. 2024).

Multivariate analyses showed that internal relative humidity was the main variable differentiating the genotypes; however, this result should not be interpreted in isolation. Because the relative humidity depends on temperature (Human et al. 2006), elevated internal relative humidity values represent the operational trace of evaporative thermolysis performed by bees to maintain an adequate thermal range (Ostwald et al. 2016; Kutby et al. 2024). Thus, discrimination based on humidity does not mean that humidity itself is the determining factor of differentiation; rather, it indicates that each genotype responds to thermal stress with distinct adaptive strategies.

Africanized honey bees, which are better adapted to hot and dry climates, exhibit greater thermal stability and lower coupling between internal temperature and internal relative humidity. This suggests that they likely first relied on the ventilation mechanism and subsequently on evaporative cooling associated with ventilation. Domingos et al. (2022) observed that Africanized honey bees select the cooling strategy according to temperature and solar radiation (which also contributes to colony overheating). These authors also reported that during the hottest hours of the day, ventilation activity was low even under extreme heat, as the bees opted for more efficient strategies, namely, those involving the use of water to cool the colony. In the present study, Africanized bees demonstrated the ability to sustain slightly higher yet more stable internal temperature values without primarily relying on evaporative cooling, which may represent an adaptive advantage.

The Buckfast bees, on the other hand, exhibited the opposite pattern. The higher internal relative humidity observed reflect a greater demand for thermoregulation, relying on evaporative cooling to compensate for their lower heat tolerance (Kutby et al. 2024). Simultaneously, the internal fluctuation pattern suggests a reduced investment in ventilation, which reinforces the strong coupling between internal temperature and internal relative humidity. The adaptive strategy used by Buckfast bees became an unfavorable trade-off in the hot and dry environment; that is, they were able to reduce temperature, but at the cost of increasing water-related stress.

Toledo and Nogueira-Couto (1999) evaluated internal temperature in Africanized honey bees and European colonies (Carniolan, Italian, and Caucasian) under tropical conditions in Brazil. They observed a internal temperature of 33 °C in all populations between 14:00 and 16:00. Abou-Shaara et al. (2012) found that Yemeni bees are more tolerant than Carniolans under extreme temperature conditions.

The assessment of the 24-hour thermo-hygrometric cycle clearly highlighted these differences. Although both populations maintained their IT within the acceptable range of 32–36 °C (Jones and Oldroyd 2006; Bonoan et al. 2014) during the heat peak, particularly between 12:00 and 16:00, they did so using different behavioral strategies. Africanized bees sustained this balance for a longer period and with lower variability in the internal relative humidity, whereas Buckfast bees responded more abruptly, exhibiting more intense oscillations, as if they were continuously reacting to the thermal environment rather than buffering it.

It is important to emphasize that maintaining temperature within tolerable limits occurs at the expense of substantial behavioral plasticity, which may vary among different groups of honeybees. For example, Gardner et al. (2007) observed that increased demand for thermoregulatory activity did not lead to a higher recruitment of workers; instead, certain “thermal specialist” workers intensified their efforts rather than redistributing tasks within the colony. In contrast, Vogt (1986a) reported that the number of bees recruited to maintain the internal temperature may vary according to the colony’s thermal demand, indicating greater flexibility in worker allocation. In both scenarios, this represents a potential cost to the colony, as workers engaged in homeostatic tasks remain stationary and are therefore unable to perform other essential tasks.

The interpretation of the data becomes more sustainable when seasonal transitions are considered. At the end of the rainy period in July, the ambient air became drier. This moment represents a critical point of ecological inflection: as thermal and water demand increases more intensely, physiological differences between the genotypes studied are amplified, and exposure to continuous thermal stress may affect the survival of bees (Kovac et al. 2014; Li et al. 2019). The Africanized honey bees maintained thermo-hygrometric regulation, whereas Buckfast operated at the physiological limit and, as a consequence, collapsed completely by the first half of August, despite constant shading and controlled management conditions.

This generalized mortality demonstrates that the problem was not merely environmental but involved multiple stressors acting in an integrated manner on colony physiology, resulting in the structural incapacity of Buckfast bees to cope with a dry tropical climate. These stressors include high thermal load, low relative air humidity, water restriction, increased energetic costs associated with thermoregulation, and metabolic stress related to maintaining brood homeostasis. The results indicate that resilience in this environment depends on the combined effects of thermal tolerance, evaporative efficiency, coordinated ventilation, water economy, and behavioral adjustment. Africanized bees appear to balance these functions more stably, whereas Buckfast bees rely more heavily on evaporation, which is a physiologically costly process for bees. In contrast, Buckfast colonies showed a greater dependence on evaporative thermoregulation, a more energetically demanding mechanism that increases water consumption, elevates worker energy expenditure, and exacerbates stress under dry conditions, thereby contributing to the observed population collapse.

Toledo and Nogueira-Couto (1999) compared thermoregulatory responses between Africanized and European bees, but considered only temperature as a variable. Although they observed similar thermal maintenance between the groups, the authors did not evaluate other critical environmental stressors such as relative humidity, ventilatory effort, or physiological costs of evaporation. Moreover, it should be considered that, in that study, the workers responsible for thermoregulation were Africanized, since the European queens had been inseminated by Africanized drones. This limits the direct attribution of thermoregulatory efficiency to the European lineage and reinforces the importance of genetic components in adaptation to tropical environments.

Although there are relevant contributions in the literature, this study advances the understanding of the thermo-hygrometric dynamics of these bees by considering not only environmental patterns but also daily evaluation cycles. The association of all evaluated aspects, including the comparison between groups, is essential for understanding the factors that shape thermal balance inside the hive.

This study identified distinct adaptive strategies employed by Africanized and Buckfast bees. Buckfast bees exhibited a more intense reliance on evaporative heat dissipation, with lower thermal stability, indicating a reduced heat tolerance. In contrast, Africanized bees maintained thermal stability with smaller fluctuations in humidity, primarily through ventilation and, subsequently, evaporative cooling associated with ventilation. These results reinforce that, in regions with a dry tropical climate, physiological and behavioral plasticity in response to continuous thermal stress is a key determinant of the survival of bee colonies. Future studies should integrate these variables with productive performance analyses and further investigate this dynamic in colonies exposed to direct solar radiation, a condition that is frequently encountered in semi-arid environments.

## Data Availability

The datasets generated and/or analyzed during the current study have not been previously published and are available in this manuscript. Any clarifications can be requested from the corresponding author.

## Declarations

This manuscript does not contain any clinical studies or patient data.

## References

[CR1] Abou-Shaara HF, Al-Ghamdi AA, Mohamed AA (2012) Tolerance of two honey bee races to various temperature and relative humidity gradients. Env Exp Biol 10:133–138

[CR2] Abou-Shaara HF, Owayss AA, Ibrahim YY, Basuny NK (2017) A review of impacts of temperature and relative humidity on various activities of honey bees. Insect Soc 64:455–463. 10.1007/s00040-017-0573-8

[CR3] Bonoan RE, Goldman RR, Wong PY, Starks PT (2014) Vasculature of the hive: heat dissipation in the honey bee (*Apis mellifera*) hive. Naturwissenschaften 101:459–465. 10.1007/s00114-014-1174-2 24760416 10.1007/s00114-014-1174-2

[CR4] Domingos HGT, Gramacho KP, Gonçalves LS (2022) Controle de temperatura em colônias de abelhas africanizadas (*Apis mellifera L*.) em diferentes faixas de temperatura do ar e radiação solar. São Paulo, São Paulo

[CR5] Domingos HGT, Sombra DS, Santos RG, Gramacho KP, Gonçalves LS (2018) Surface temperature and heat transfer between body regions of Africanized honeybees (*Apis mellifera* L.) in hives under sun and shade conditions in the Northeastern Semi-arid Region of Brazil. J Agric Sci Technol 8:28–35. 10.17265/2161-6256/2018.01.004

[CR6] Gardner KE, Foster RL, O’Donnell S (2007) Experimental analysis of worker division of labor in bumblebee nest thermoregulation (*Bombus huntii*, Hymenoptera: Apidae). Behav Ecol Sociobiol 61:783–792. 10.1007/s00265-006-0309-7

[CR7] Gonzalez VH, Herbison N, Robles Perez G, Panganiban T, Haefner L, Tscheulin T, Petanidou T, Hranitz J (2024) Bees display limited acclimation capacity for heat tolerance. Biol Open 13:1–8. 10.1242/bio.060179 38427330 10.1242/bio.060179 PMC10979511

[CR8] Human H, Nicolson SW, Dietemann V (2006) Do honeybees, *Apis mellifera scutellata*, regulate humidity in their nest? Naturwissenschaften 93:397–401. 10.1007/s00114-006-0117-y 16670906 10.1007/s00114-006-0117-y

[CR9] Jones JC, Oldroyd BP (2006) Nest thermoregulation in social insects. Adv Insect Physiol 33:153–191. 10.1016/S0065-2806(06)33003-2

[CR10] Kovac H, Käfer H, Stabentheiner A, Costa E (2014) Metabolism and upper thermal limits of *Apis mellifera carnica* and *A. m. ligustica*. Apidologie 45:664–677. 10.1007/s13592-014-0284-3 25378763 10.1007/s13592-014-0284-3 PMC4218932

[CR11] Kühnholz S, Seeley TD (1997) The control of water collection in honey bee colonies. Behav Ecol Sociobiol 41:407–422

[CR12] Kutby R, Baer-Imhoof B, Robinson S, Porter L, Baer B (2024) The effect of hive type on colony homeostasis and performance in the honey bee (*Apis mellifera*). Insects 15:1–16. 10.3390/insects15100800 10.3390/insects15100800 PMC11508670 39452376

[CR13] Li X, Ma W, Shen J, Long D, Feng Y, Su W, Xu K, Du Y, Jiang Y (2019) Tolerance and response of two honeybee species *Apis cerana* and *Apis mellifera* to high temperature and relative humidity. PLoS One 14:e0217921. 10.1371/journal.pone.0217921 31170259 10.1371/journal.pone.0217921 PMC6553758

[CR14] Ma CS, Ma G, Pincebourde S (2021) Survive a warming climate: insect responses to extreme high temperatures. Annu Rev Entomol 66:163–184. 10.1146/annurev-ento-041520-074454 32870704 10.1146/annurev-ento-041520-074454

[CR15] Nelson RM, Wallberg A, Simões ZLP, Lawson DJ, Webster MT (2017) Genomewide analysis of admixture and adaptation in the Africanized honeybee. Mol Ecol 26:3603–3617. 10.1111/mec.14122 28378497 10.1111/mec.14122

[CR16] Ostwald MM, Smith ML, Seeley TD (2016) The behavioral regulation of thirst, water collection and water storage in honey bee colonies. J Exp Biol 219:2156–2165. 10.1242/jeb.139824 27445400 10.1242/jeb.139824

[CR17] Poot-Báez V, Medina-Hernández R, Medina-Peralta S, Quezada-Euán JJG (2020) Intranidal temperature and body size of Africanized honey bees under heatwaves (Hymenoptera: Apidae). Apidologie 51:382–390. 10.1007/s13592-019-00725-5

[CR18] Potts SG, Imperatriz-Fonseca V, Ngo HT, Aizen MA, Biesmeijer JC, Breeze TD, Dicks LV, Garibaldi LA, Hill R, Settele J, Vanbergen AJ (2016) Safeguarding pollinators and their values to human well-being. Nature 540:220–229. 10.1038/nature20588 27894123 10.1038/nature20588

[CR19] Santos RG, Domingos HGT, Silva LA, Sombra DS, Gramacho KP, Gonçalves LS (2023) Comparative study of the performance of Africanized bees managed in thermal stress and thermal comfort in a semiarid region. AVB 17:80–85. 10.21708/avb.2023.17.4.12083

[CR20] Silva LA, Silva AD, Domingos HGT, Bergamo GC, Message D, Gramacho KP (2024) *Varroa destructor* mite population dynamics in Africanized honeybee (*Apis mellifera*) colonies in a semi-arid region. Exp Appl Acarol 93:537–547. 10.1007/s10493-024-00944-1 38985397 10.1007/s10493-024-00944-1

[CR21] Simpson J (1991) Nest climate regulation in honey bee colonies. Science 133:1327–1333 10.1126/science.133.3461.1327 17744947

[CR22] Toledo VDAAD, Nogueira-Couto RH (1999) Thermoregulation in colonies of Africanized and hybrids with Caucasian, Italian and Carniolan *Apis mellifera* honey bees. Braz Arch Biol Technol 42:1–7. 10.1590/S1516-89131999000400007

[CR23] Vogt FD (1986a) Thermoregulation in bumblebee colonies. I. Thermoregulatory versus brood-maintenance behaviors during a cute changes in ambient temperatures. Physiol Zool 59:55–59

[CR24] Winston ML (1991) The biology of the honey bee. Harvard University Press

